# Antagonistic effect of TNF-alpha and insulin on uncoupling protein 2 (UCP-2) expression and vascular damage

**DOI:** 10.1186/s12933-014-0108-9

**Published:** 2014-07-31

**Authors:** Almudena Gómez-Hernández, Liliana Perdomo, Natalia de las Heras, Nuria Beneit, Óscar Escribano, Yolanda F Otero, Carlos Guillén, Sabela Díaz-Castroverde, Beatriz Gozalbo-López, Victoria Cachofeiro, Vicente Lahera, Manuel Benito

**Affiliations:** Biochemistry and Molecular Biology Department, School of Pharmacy, Complutense University of Madrid, Madrid, Spain; CIBER of Diabetes and Associated Metabolic Diseases, Madrid, Spain; Almudena Gómez-Hernández, Biochemistry and Molecular Biology Department, School of Pharmacy, Complutense University of Madrid, Madrid, 28040 Spain; Physiology Department, School of Medicine, Complutense University of Madrid, Madrid, Spain

**Keywords:** TNF-α, Insulin, UCP-2, Atherogenesis

## Abstract

**Background:**

It has been reported that increased expression of UCP-2 in the vasculature may prevent the development of atherosclerosis in patients with increased production of reactive oxygen species, as in the diabetes, obesity or hypertension. Thus, a greater understanding in the modulation of UCP-2 could improve the atherosclerotic process. However, the effect of TNF-α or insulin modulating UCP-2 in the vascular wall is completely unknown. In this context, we propose to study new molecular mechanisms that help to explain whether the moderate hyperinsulinemia or lowering TNF-α levels might have a protective role against vascular damage mediated by UCP-2 expression levels.

**Methods:**

We analyzed the effect of insulin or oleic acid in presence or not of TNF-α on UCP-2 expression in murine endothelial and vascular smooth muscle cells. At this step, we wondered if some mechanisms studied in vitro could be of any relevance in vivo. We used the following experimental models: ApoE^−/−^ mice under Western type diet for 2, 6, 12 or 18 weeks, BATIRKO mice under high-fat diet for 16 weeks and 52-week-old BATIRKO mice with o without anti-TNF-α antibody pre-treatment.

**Results:**

Firstly, we found that TNF-α pre-treatment reduced UCP-2 expression induced by insulin in vascular cells. Secondly, we observed a progressive reduction of UCP-2 levels together with an increase of lipid depots and lesion area in aorta from ApoE^−/−^ mice. In vivo, we also observed that moderate hyperinsulinemic obese BATIRKO mice have lower TNF-α and ROS levels and increased UCP-2 expression levels within the aorta, lower lipid accumulation, vascular dysfunction and macrovascular damage. We also observed that the anti-TNF-α antibody pre-treatment impaired the loss of UCP-2 expression within the aorta and relieved vascular damage observed in 52-week-old BATIRKO mice. Finally, we observed that the pretreatment with iNOS inhibitor prevented UCP-2 reduction induced by TNF-α in vascular cells. Moreover, iNOS levels are augmented in aorta from mice with lower UCP-2 levels and higher TNF-α levels.

**Conclusions:**

Our data suggest that moderate hyperinsulinemia in response to insulin resistance or lowering of TNF-α levels within the aorta attenuates vascular damage, this protective effect being mediated by UCP-2 expression levels through iNOS.

**Electronic supplementary material:**

The online version of this article (doi:10.1186/s12933-014-0108-9) contains supplementary material, which is available to authorized users.

## Background

Uncoupling proteins (UCPs) belong to the family of mitochondrial transporter proteins and are important for lowering mitochondrial membrane potential and dissipating metabolic energy as heat, maintenance of respiration, glucose disposal rate, insulin secretion, prevention of reactive oxygen species (ROS) production [1,2]. UCP-1 was the first member identified, expressed primarily in brown adipose tissue and the major contributor to energy expenditure [3]. Other four members of UCP (−2 to −5) family have been indentified. In contrast to UCP-4 and −5, human UCP-2 and −3 are both more closely related to UCP-1 [4,5]. UCP-2 is expressed widely and in human is highly expressed in white adipose tissue. Other tissues as skeletal muscle, heart, cell of immune system and vascular cells express considerable amounts of UCP-2 [6]. Recent studies from UCP-2 and −3 knockout mice suggest that both UCPs have uncoupling activity and decreased ROS production in macrophages and skeletal muscle, respectively [7–9]. More recently, a direct role for UCP-2 in the regulation of atherogenesis has been suggested by the observation that bone marrow transplantation from UCP-2-deficient mice to LDLR^−/−^ mice markedly increased atherosclerotic lesion size [10]. Moreover, it has been described that UCP-2 overexpression in the vasculature may prevent the development of atherosclerosis in patients with increased ROS, such as in diabetes, obesity or hypertension [11] and ameliorate hyperglycemia-induced endothelial dysfunction [12]. Furthermore, UCP-2 might be playing an important role in the regulation of energy expenditure and are likely to contribute to obesity and type 2 diabetes mellitus (T2DM). In this regard, several UCP-2 gene polymorphisms were linked to an increased body weight index or obesity in Pima Indians [13,14] and in Balinese population [15] or with insulin resistance or T2DM [16–18]. Thus, reduced UCP gene expression has been found in adipose tissue of obese subjects and in the first-degree relatives of T2DM patients. On the other hand, both obese and diabetic patients have associated vascular complications such as atherosclerosis [19,20], insulin resistance with hyperinsulinemia and elevated circulating TNF-α levels [21]. To get a new insight on that UCP-2 protective effect on the vasculature, we have studied new molecular mechanisms that help to explain whether the moderate hyperinsulinemia or reduction TNF-α levels might have a protective role against vascular damage mediated by UCP-2 modulation. Firstly, we have analyzed the effect of insulin and/or TNF-α on UCP-2 levels in endothelial and vascular smooth muscle cells. After that, we wondered if some mechanisms studied in vitro could be of any relevance in vivo. We used the following experimental models: ApoE^−/−^ mice at 8, 12, 18 or 24 weeks of age, BATIRKO mice under high-fat diet for 16 weeks and 52-wk-old BATIRKO mice with o without anti-TNF-α treatment to address the relationship between UCP-2 expression, or lipid accumulation, or vascular damage, or oxidative stress, or insulin or TNF- α plasma levels. Finally, we searched the role of iNOS in the inhibition of UCP-2 expression by TNF-α.

## Methods

### Cell culture

Primary vascular smooth muscle cells (VSMC) were obtained from thoracic aorta arteries, immortalized and cultured as described previously [22]. Endothelial cell line, SVEC4-10EE2 (clone 2167) was purchased from ATCC and was cultured in DMEM medium supplemented with 10% of horse bovine serum, respectively. Both cell lines were growth-arrested by incubation in medium without serum for 5 h, and then incubated with the corresponding stimuli. For experiments in vitro, we have used TNF-α (10 ng/mL), insulin (10 nmol/L), oleate (1 mmol/L) and L-NAME (1 mmol/L).

### Experimental models

Male mice were maintained in the Animal Care Facility under the standard conditions of temperature and 12 h light/dark cycle. All animals from three experimental models used are under C57BL/6 genetic background. Male ApoE^−/−^ knockout mice and their control mice were fed a Western type diet (A04 + 21% kcal from fat) at six week-age for 2, 6, 12 or 18 weeks respectively. Male BATIRKO mice [22] were fed on high-fat diet (A04 + 61% kcal from fat) for 16 weeks or standard diet (3% calories from fat, A04) for 52 weeks. Moreover, one group of 52-wk-old BATIRKO mice were treated with LEAF purified anti-TNF-α (MP6-XT22, Bio-Legend, San Diego, CA) (50 μg/mouse ip.) every 3 days for 6 weeks as previously described [23]. All animal experimentation described in this manuscript was conducted according with accepted standards of human animal care, as approved by the corresponding institutional committee. The investigation also conforms to the Guide for the Care and Use of Laboratory Animals published by the National Institutes of Health (NIH Publication No. 85–23, revised 1996) and in accordance with The ARRIVE Guideline for Reporting Animal research [24].

### Western blot

Western blot analyses were performed on protein extracts from VSMCs, ECs or aorta artery as previously described [25]. The antibodies used were anti-phospho-AKT (T308), AKT, p-p70S6K (T389), p70S6K, p-p44/42 (S202/T204) and p44/42 from Cell Signalling, anti-UCP-2 was from Calbiochem and anti-β-actin or α-tubulin was from Sigma-Aldrich Corp.

### RNA extraction and real-time quantitative PCR

Total RNA was extracted from ECs, VSMCs or aorta artery from mice by TRIzol method (Invitrogen, Carlsbad, CA). The gene expression was analyzed by real-time quantitative PCR (qRT-PCR) as described [25].

### Analytical procedures

Plasma levels of insulin and TNF-α were analyzed using ELISA kits (Millipore and SABioSciences, Frederick, MD, respectively).

### Histological analysis

Aortic roots were OCT-embedded and sections of 7 μm interval were Oil-Red-O/hematoxylin stained was done to measure lipid depot. The lesion size on aortic root was also measured as described [22]. Macrophages and nitrotyrosine levels were detected by immunoperoxidase with rat anti-mouse F4/80 antigen (MCA497GA, AbD serotec) and rabbit anti-nitrotyrosine polyclonal Ab (06–284, Upstate), respectively.

### Statistical analysis

All values were expressed as means +/−sem. Data were analyzed using a one-way analysis of variance, followed by a Bonferroni test if differences were noted (SPSS 15.0 program). Spearman’s correlation coefficient analysis was used to assess associations between several parameters of experimental model. The null hypothesis was rejected when the p value was less than 0.05.

## Results

### Differential effect of TNF-alpha and Insulin on UCP-2 expression in vascular cells

**Figure 1:**
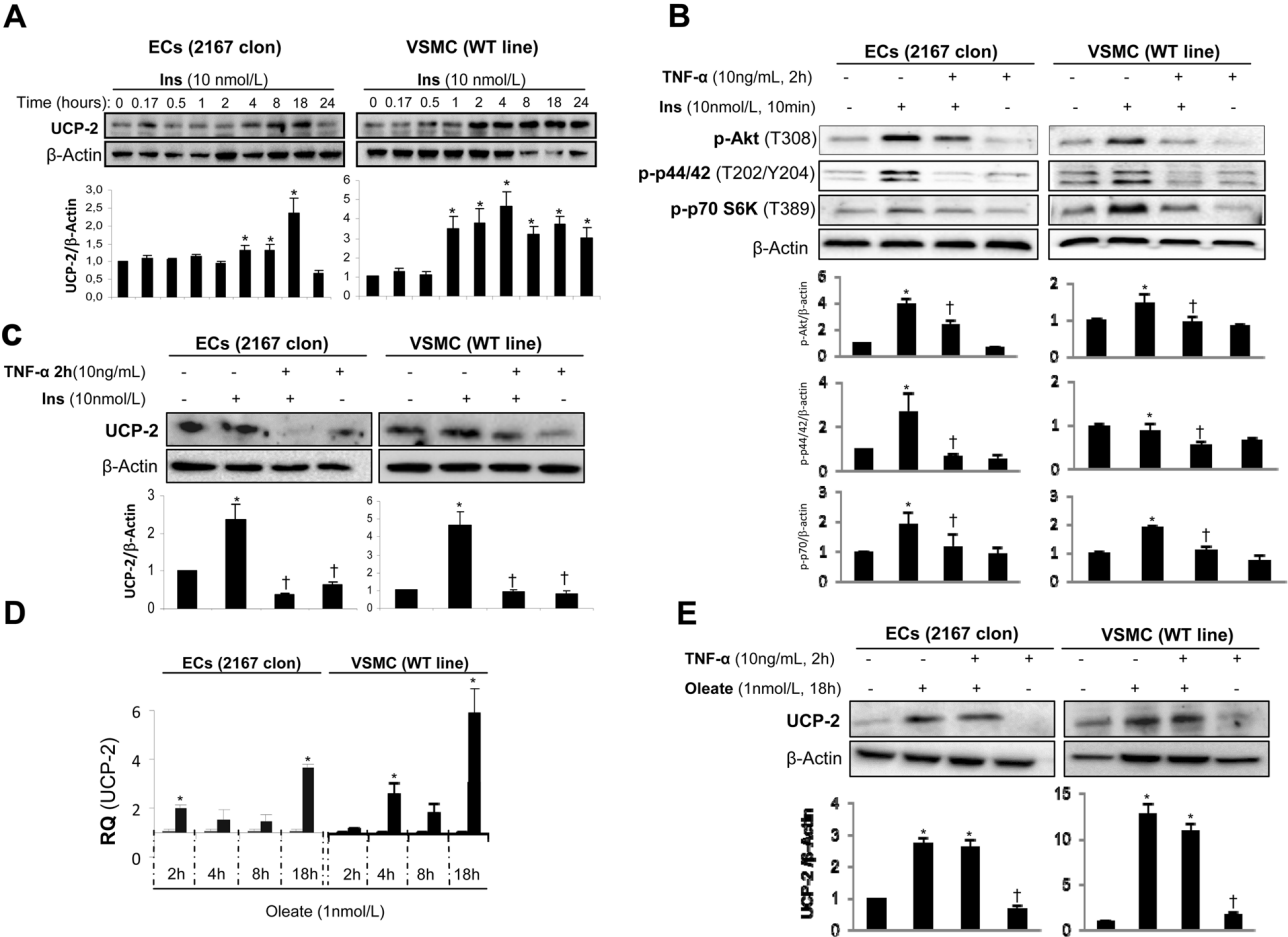
**Effect of insulin, oleate and TNF-α on UCP-2 expression level in vascular cells. (A)** The effect of insulin on UCP-2 expression levels in ECs and VSMCs was analyzed by Western blot. **(B)** Effect of TNF-α on the phosphorylation of Akt, p44/42 and p70S6K in both vascular cell lines stimulated by insulin. **(C)** Effect of TNF-α pretreatment on UCP-2 protein levels in vascular cell lines stimulated by insulin. **(D)** qRT-PCR analysis of UCP-2 mRNA expression in vascular cell lines stimulated by oleate. **(E)** Effect of TNF-α pretreatment on UCP-2 expression in vascular cell lines stimulated by oleate. β-Actin was used as loading control. *p < 0.05 vs. control; † p < 0.05 vs. stimulus.

Although it is well-known the protective role of UCP-2 against vascular damage [10], the relationship between insulin or TNF-α with UCP-2 in vascular cells is completely unknown. Thus, we addressed that issue in ECs and VSMCs as two major components of the vascular wall. Firstly, we observed that insulin significantly increased UCP-2 protein levels at 4, 8 and 18 h in ECs and at 1 h through 24 h in VSMCs (Figure 1A). We ascertained that the pre-treatment with 10 ng/mL TNF-α for 2 hours induced insulin resistance in both vascular cells as shown by the significant decreases in the phosphorylation of Akt, p70S6K and p44/42 in cells stimulated with 10 nmol/L insulin for 10 minutes (Figure 1B). Based on these data, we analyzed the effect of TNF-α on UCP-2 protein levels. Thus, 10 ng/mL TNF-α for 2 hours downregulated UCP-2 protein levels in both vascular cells respectively (Figure 1C and E). More importantly, we also demonstrated that TNF-α pre-treatment induced a significant decrease of UCP-2 protein levels in VSMCs and ECs stimulated upon insulin action for 4 h or 18 h, respectively (Figure 1C). At this stage, we compared insulin effect with a well-known inductor of UCP-2 expression such as oleate [26]. Firstly, we observed a significant increase in UCP-2 expression at mRNA or protein level at 18 h upon oleate treatment in both vascular cell lines respectively (Figure 1D and E). However, TNF-α pre-treatment did not significantly impair UCP-2 protein induced by oleate in both vascular cells respectively (Figure 1E).

### Protective role of UCP-2 against lipid accumulation and vascular damage

**Figure 2:**
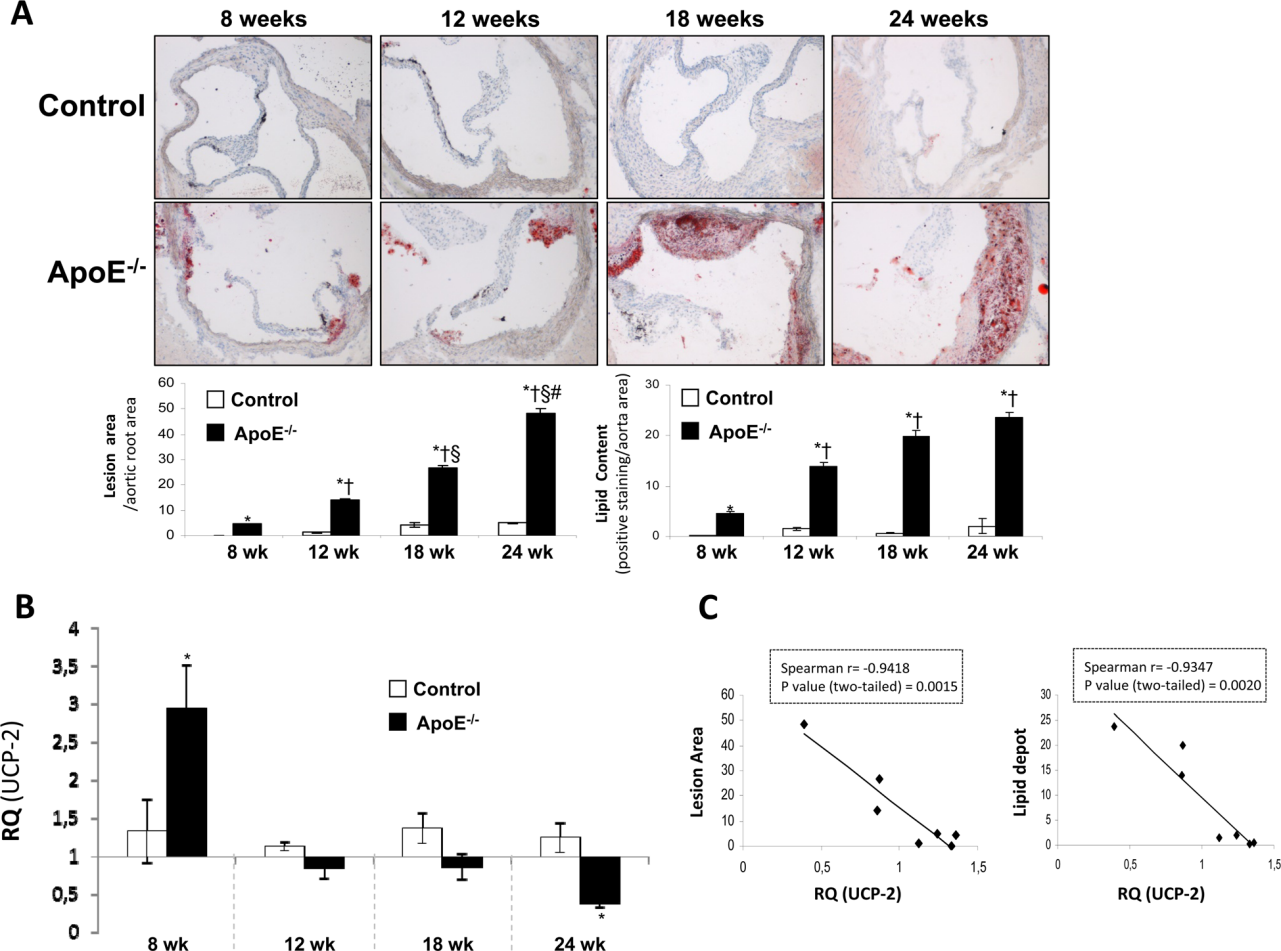
**Role of UCP-2 in the aorta from Control and ApoE** ^**−/−**^ **mice. (A)** Representative photomicrographs of OilredO staining of aortic roots and quantification of lesion area and lipid content from Control and ApoE^−/−^ mice at 8, 12, 18 or 24 weeks of age. **(B)** qRT-PCR analysis of UCP-2 mRNA expression in aorta artery. **(C)** Spearman correlation between UCP-2 expression levels in the aorta by qRT-PCR and lesion area or lipid depot. Control at 8 weeks (Control 8wk, n = 3); ApoE^−/−^ at 8 weeks (ApoE^−/−^ 8wk, n = 3); Control at 12 weeks (Control 12wk, n = 5); ApoE^−/−^ at 12 weeks (Control 12wk, n = 4); Control at 18 weeks (Control 18wk, n = 4); ApoE^−/−^ at 18 weeks (ApoE^−/−^ 18wk, n = 6); Control at 24 weeks (Control 24wk, n = 5); ApoE^−/−^ at 24 weeks (ApoE^−/−^ 24wk, n = 5). *p < 0.05 vs. each control; †p < 0.05 vs. ApoE^−/−^ 8wk; §p < 0.05 vs. ApoE^−/−^ 12wk; #p < 0.05 vs. ApoE^−/−^ 18wk.

At this step, we wondered if some mechanisms described in vitro could be of any relevance in vivo. Thus, we explored in vivo the associations between UCP-2 levels and lipid accumulation, or vascular damage, or oxidative stress, or TNF-α levels in the aortic wall, or with circulating insulin levels. We used different mouse models of disease. The first one was ApoE^−/−^ mice and their controls at 8, 12, 18 and 24 wk of age. By Oil-Red-O/hematoxylin staining, we observed that ApoE^−/−^ mice developed progressively higher lesion area with higher lipid content within aortic roots (Figure 2A). We also analyzed by qRT-PCR UCP-2 mRNA expression and its likely relationship with the lesion area or lipid content. Thus, UCP-2 levels augmented significantly within the aorta from 8 week-old ApoE^−/−^ mice as compared with their corresponding controls (Figure 2B). However, UCP-2 levels sharply decreased from 12- through 24-wk-old ApoE^−/−^ mice (Figure 2B). Thus, we established a negative and significant correlationship between UCP-2 levels and lesion area or lipid depot within the aortic wall (Figure 2C). Additionally, we checked that UCP-2 protein levels were also decreased in 24-wk-old ApoE^−/−^ mice in relation to theirs controls (Additional file 1: Figure S1A).

**Figure 3:**
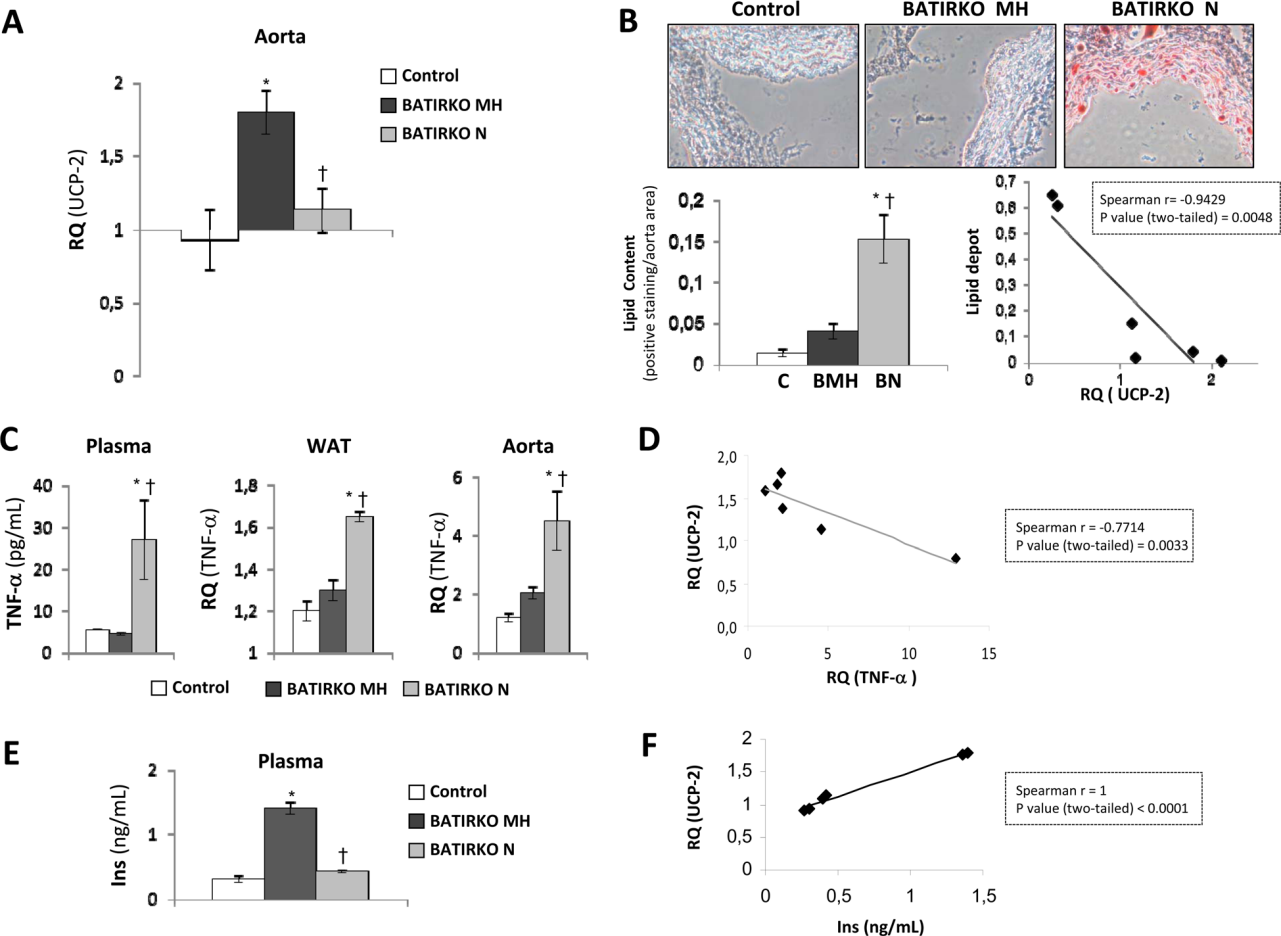
**Role of UCP-2 in the aorta from Control and BATIRKO mice under high-fat diet. (A)** qRT-PCR analysis of UCP-2 expression levels in aorta from Control and BATIRKO mice under HFD. **(B)** Representative photomicrographs of OilredO staining of aortic roots from Control and BATIRKO under high-fat diet and quantification of lipid content and its correlation with UCP-2 expression levels in the aorta. **(C)** Determination of TNF-α plasma levels (by ELISA) and in WAT and aorta (by qRT-PCR). **(D)** Spearman correlation between UCP-2 and TNF-α levels in the aorta by qRT-PCR. **(E)** Plasma insulin levels by ELISA in Control and BATIRKO under high-fat diet. **(F)** Spearman correlation between UCP-2 expression levels in aorta and plasma insulin levels. Control under HFD (Control; n = 12); moderate hyperinsulinemic obese BATIRKO mice (BATIRKO MH, n = 6); normoinsulinemic obese BATIRKO mice (BATIRKO N, n = 10). *p < 0.05 vs. Control; †p < 0.05 vs. BATIRKO MH mice.

The second mouse-model studied was BATIRKO mice under HFD for 16 weeks. These mice lacking IR in BAT-specific manner under STD showed severe brown lipoatrophy, susceptibility to the obesity (mainly in gonadal WAT compartment), glucose intolerance and a defect in insulin secretion [22,27]. Moreover, BATIRKO mice under HFD also showed insulin resistance and more severe glucose intolerance. So, we have phenotypically established two groups of obese BATIRKO mice as characterized by their plasma insulin levels, pancreatic islet area, islet insulin content, and also by their glucose tolerance curves and fasting hyperglycemia. The first group showed compensated insulin resistance (moderately hyperinsulinemic, BATIRKO MH, fasted insulin plasma levels = 1.4 ± 0.2 ng/mL, p < 0.05 vs. control group), marked pancreatic beta cell hyperplasia (0.06 ± 0.01 islet area/pancreas area, p < 0.05 vs. control group) and higher islet insulin content (0.28 ± 0.02 positive staining/ islet area, p < 0.05 vs. control group). A second group showed non-compensated insulin resistance (normoinsulinemic, BATIRKO N, fasted insulin plasma levels = 0.42 ± 0.05 ng/mL, p < 0.05 vs. BATIRKO MH), normal islet size (0.02 ± 0.008 islet area/pancreas area, ns. vs. control group) and lower islet insulin content (0.14 ± 0.01 positive staining/islet area, p < 0.05 vs. control group). In addition, normoinsulinemic obese BATIRKO mice (N) showed a more severe glucose intolerance and mild fasting hyperglycemia as compared with moderate hyperinsulinemic obese BATIRKO mice (MH) (Fasting glycemia from BATIRKO N = 138 ± 9 mg/dL, p < 0.05 vs. control group; fasting glycemia from BATIRKO MH = 123 ± 8 mg/dL and from control group = 111 ± 4 mg/dL). Under this scenario, we observed that BATIRKO MH mice showed a significant increase of UCP-2 levels as compared with control or BATIRKO N mice respectively (Figure 3A and Additional file 1: Figure S1B). At this stage, we explored the relationship between UCP-2 levels within the aortic wall and the vascular damage. Thus, normoinsulinemic obese BATIRKO mice with lower UCP-2 levels in the aorta showed endothelial dysfunction (Additional file 2: Figure S2A), higher vasoconstrictor response to angiotensin II or TXA_2_ (Additional file 2: Figure S2B), higher lipid depots (Figure 3B), higher lesion area and macrophage infiltration in aortic roots (Additional file 2: Figure S2C) as compared with moderate hyperinsulinemic BATIRKO mice. Moreover, we also observed a significant negative correlationship between UCP-2 levels and lipid depots or lesion area in the aorta in those animals (Figure 3B).

### Relationship between TNF-α and UCP-2 expression levels *in vivo*

**Figure 4:**
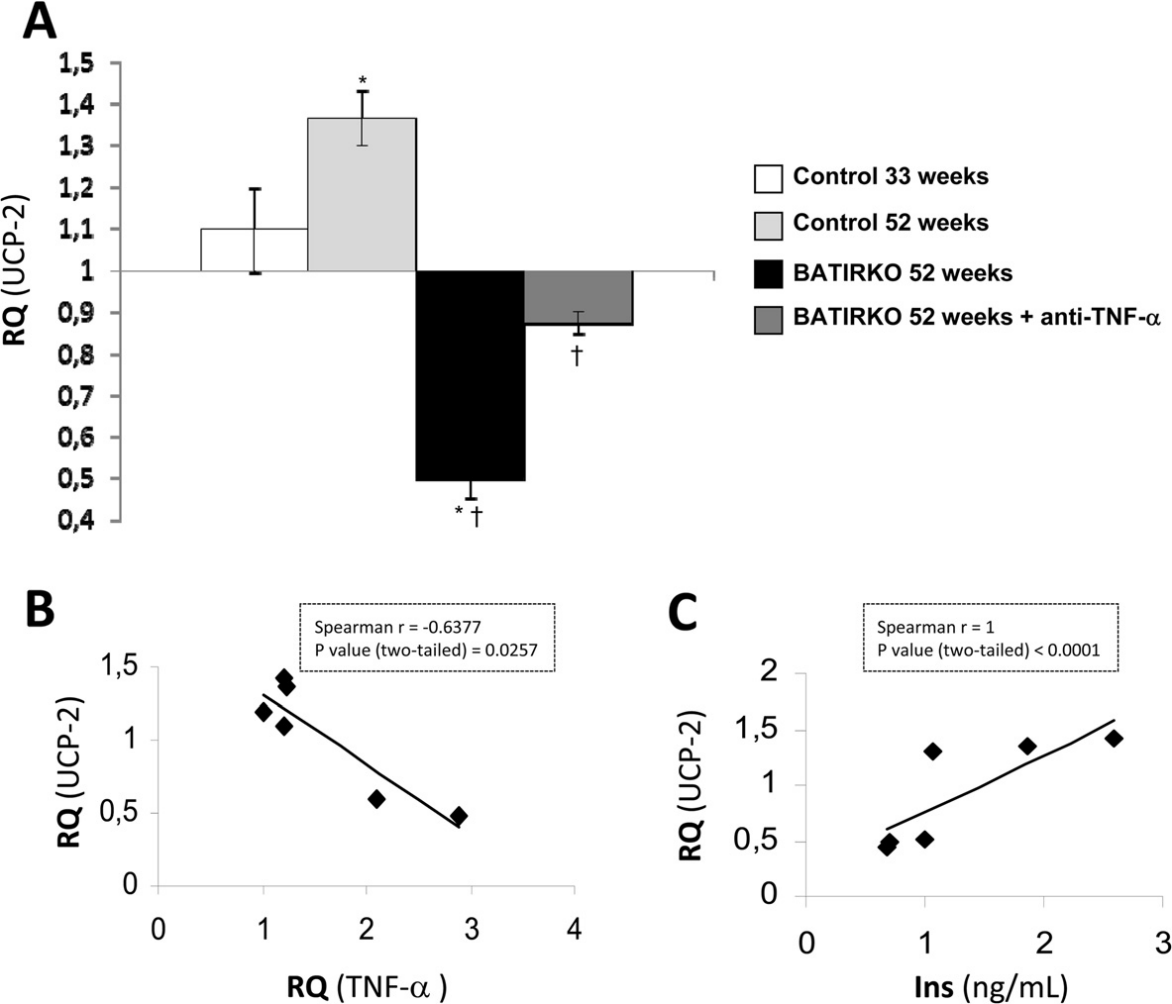
**Effect of anti-TNF-α pre-treatment on UCP-2 expression level in BATIRKO mice. (A)** qRT-PCR analysis of UCP-2 mRNA expression in aorta from Control and BATIRKO mice and anti-TNF-α treated BATIRKO mice at 52 weeks of age. Spearman correlation between UCP-2 expression levels and TNF-α in the aorta **(B)**, or with plasma insulin levels **(C)**. Control 33 weeks (n = 12); Control 52 weeks (n = 12); BATIRKO 52 weeks (n = 8); BATIRKO 52 weeks + anti-TNF-α (n = 3). *p < 0.05 vs. Control; †p < 0.05 vs. 52-wk-old-BATIRKO mice.

As we had demonstrated in vitro that TNF-α might downregulate UCP-2 protein levels in vascular cells, we explored that mechanism in both groups of obese BATIRKO mice in the aorta (Figure 3C). Normoinsulinemic obese BATIRKO mice (N) with lower UCP-2 levels in aorta and higher vascular damage showed a significant increase in TNF-α plasma levels, or expressed in WAT or aorta as compared with moderate hyperinsulinemic BATIRKO mice (MH) (Figure 3C). Thus, we observed a significant negative correlationship between UCP-2 and TNF-α levels in the aorta (Figure 3D). We also explored those levels in 52-week-old BATIRKO mice under standard diet. Those mice showed severe brown lipoatrophy, obesity, hypoinsulinemia, mild fasting hyperglycemia, glucose intolerance, vascular dysfunction, macrophage infiltration, oxidative stress, and a significant increase of gene markers of endothelial activation and inflammation as previously characterized [23], TNF-α playing a major role [23]. Now, we have observed that 52- week-old BATIRKO mice show a significant reduction of UCP-2 expression levels, that UCP-2 lowering in the aorta was precluded by the pre-treatment with anti-TNF-α (Figure 4A and Additional file 1: Figure S1C and D). In addition, a significantly negative correlationship between UCP-2 and TNF-α expression levels in the aorta was also observed (Figure 4B).

### Effect of insulin on UCP-2 expression levels *in vivo*

At this step, we wondered if UCP-2 overexpression induced by insulin in vitro could be of any relevance in vivo. For this purpose, we observed that obese BATIRKO mice with moderate hyperinsulinemia had higher UCP-2 levels in aorta and lesser vascular damage than normoinsulinemic obese BATIRKO mice (Figure 3A, B and E and Additional file 1: Figure S1B). Moreover, we established a positive and significant correlation between circulating insulin levels and UCP-2 levels in aorta (Figure 3F). In the third experimental model, we also observed this correlationship between insulin and UCP-2 expression levels (Figure 4C). Thus, 52-week-old control group displaying moderate hyperinsulinemia showed a significant increase in UCP-2 expression levels in the aorta (Figure 3A and Additional file 1: Figure S1D). However, 52-week-old BATIRKO mice showing a lower insulinemia manifested a significant reduction of UCP-2 expression levels and higher vascular alterations (Figure 3A and Additional file 1: Figure S1C and D). On the other hand, UCP-2 might modify atherosclerotic process due to the fact that elevated levels of this protein reduce ROS levels [11]. Thus, we observed a significant decrease of superoxide anion and nitrotyrosine levels in aortic roots from moderate hyperinsulinemic as compared with normoinsulinemic obese BATIRKO mice (Additional file 2: Figure S2C).

### Role of iNOS in the UCP-2 downregulation induced by TNF-α

**Figure 5:**
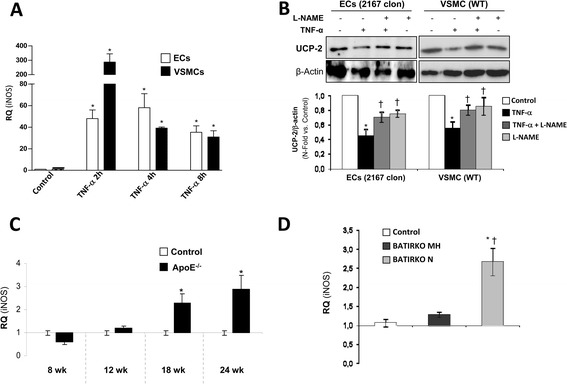
**Role of iNOS in the inhibition of UCP-2 expression level induced by TNF-α. (A)** qRT-PCR analysis of iNOS mRNA expression in ECs and VSMCs stimulated with TNF-α for 2, 4 and 8 hours. **(B)** Western blot analysis of UCP-2 levels with or without iNOS inhibitor (L-NAME) in ECs and VSMCs stimulated with TNF-α. *p < 0.05 vs. control; †p < 0.05 vs. stimuli. qRT-PCR analysis of iNOS mRNA expression in aorta from Control and ApoE^−/−^ mice at 8, 12, 18 and 24 wk of age **(C)** and Control and BATIRKO mice under high-fat diet **(D)**. *p < 0.05 vs. Control; †p < 0.05 vs. BATIRKO MH mice.

Back to in vitro studies, we explored whether the reduction in UCP-2 levels in the aorta induced by TNF-α might be mediated at least in part by iNOS in vascular cells. Thus, we observed by qRT-PCR that TNF-α treatment for 2, 4 and 8 hours induced a significant robust increase of iNOS mRNA expression in vascular cells (Figure 5A). Next step, we observed that the pre-treatment with iNOS inhibitor (L-NAME) impaired that rising of the UCP-2 expression levels in ECs or VSMCs in response to TNF-α (Figure 5B). These data strongly suggest that TNF-α downregulates UCP-2 expression levels through iNOS expression in the aortic wall. We explored that relationship between iNOS and UCP-2 *in vivo*. Thus, we observed that 18-week-old and mainly 24-week-old ApoE^−/−^ mice showed a significant increase of iNOS expression levels together with a significant reduction of UCP-2 levels in the aorta (Figure 5C). Moreover, normoinsulinemic obese BATIRKO mice with lower UCP-2 levels and higher vascular damage showed a significant increase of iNOS levels as compared with moderate hyperinsulinemic BATIRKO mice in the aorta (Figure 5D).

## Discussion

### Protector role of UCP-2 against lipid depot and vascular damage

Atherosclerosis is a multi-factorial chronic vascular inflammatory disease characterized by endothelial dysfunction and accumulation of lipids, inflammatory cells, smooth muscle cells and extracellular matrix in the arterial neointima [28]. Several studies suggest that ROS are involved in plaque formation [29] and all plaque cellular components may respond to and be damaged by ROS, contribute to plaque progression and finally, to plaque rupture [30]. Thus, several approaches to stop ROS production and to alter disease progression have been used [31,32]. In addition, it has been previously published that UCP-2 overexpression in macrophages decreases intracellular ROS levels and reduces their immune activity [33,34]. Moreover, UCP-2 might function as an adaptive antioxidant defense to protect against the development of atherosclerosis in response to high fat and cholesterol diet [35] and improve hyperglycemia-induced endothelial dysfunction [12]. Under this scenario, our results demonstrate that high-fat diet BATIRKO mice showing lower UCP-2 expression levels manifested higher oxidative stress in the aorta. Moreover, the decrease in UCP-2 levels in the aorta is strongly inversely correlated with lipid accumulation and lesion area from 24-week-old ApoE^−/−^ mice or normoinsulinemic BATIRKO mice in the aorta. Previous results have also suggested a protective role of UCP-2 against atherosclerosis [10] showing an antiatherogenic effect in macrophages, ECs and VSMCs [11]. Thus, UCP-2 higher expression reduced proliferation, migration and plasminogen activator 1 expression in human VSMCs [36].

### Insulin induces UCP-2 overexpression in aorta protecting against vascular damage

A better knowledge of UCP-2 expression levels regulation in the vasculature may improve the management of the atherosclerotic process. Thus, we explored the association between insulin and UCP-2 *in vivo* and *in vitro*. Our results suggest that insulin or moderate hyperinsulinemia in response to insulin resistance induces UCP-2 expression in ECs and VSMCs or in the aorta from BATIRKO MH mice respectively. On this regard, we previously demonstrated that insulin or IGF-1 induce UCP-1 expression through IRS-1 or AP-1 activity in a PI3K/Akt dependent manner [37,38]. Others authors had also described similar effects of insulin on UCP-2 expression levels in bovine retinal microvascular endothelial cells [39] or in skeletal muscle [40]. Moreover, it have been described that intensive insulin therapy suppressed iNOS gene expression in liver and skeletal muscle, possibly in part via reduced NF-κB activation, and lowered the elevated circulating NO levels [41]. So, insulin might also reduced NF-κB activation and iNOS levels in aorta and in consequence favours UCP-2 overexpression and protect against vascular damage.

### TNF-α downregulates UCP-2 in aorta accelerating vascular damage

Among several proinflammatory and proatherogenic signals working on the vasculature TNF-α is relevant the most. Thus, the relationship between TNF-α and UCP-2 expression levels appears to be of importance in assessing vascular damage risk. On this regard, we have shown that insulin and TNF-α have antagonistic effect on UCP-2 expression in ECs and VSMCs. It has been previously published that proinflammatory cytokines such as TNF-α and/or IL-1β downregulated UCP-2 levels in adipocytes [42], INS-1 cells or rat pancreatic islets [43]. Moreover, our data provide a strong support *in vivo* to the negative relationship between TNF-α and UCP-2. Thus, 52-week-old BATIRKO mice or normoinsulinemic BATIRKO mice under high-fat diet with lower UCP-2 levels showed elevated TNF-α expression levels in WAT, plasma and aorta. Moreover, TNF-α may directly downregulates adiponectin [44] contributing to the development of vascular insulin resistance and the decrease of UCP-2 levels in the aorta. On this regard, it has previously been described that adiponectin induces UCP-2 expression in the liver [45]. In the two populations of BATIRKO mice, we observed a negative correlation between TNF-α and adiponectin levels in both WAT and plasma. Therefore, higher levels of adiponectin might induce UCP-2 overexpression in the aorta attenuating vascular damage. The use of the anti-TNF-α antibody pre-treatment support the concept that TNF-α downregulates UCP-2 expression levels as shown in 52-week-old BATIRKO mice.

Other mechanism involved in the inhibitory effect of TNF-α on UCP-2 expression levels is the NO-dependent pathway induction of iNOS expression in ECs and VSMCs as previously described in 3T3F442A preadipocytes [42]. In vivo, we also demonstrated that anti-TNF-α treatment in 52-week-old BATIRKO mice is able to reduce NF-κB activation in white and brown adipose tissues and aorta, reducing iNOS levels in aorta [24] and increasing UCP-2 levels in aorta and as result lowering vascular damage. Moreover, LPS promoted the expression of iNOS and ROS production as well as inflammatory cytokines in UCP-2^−/−^ macrophages [46,47]. Our data strongly suggest an inverse correlationship between iNOS and UCP-2. Thus, 24-week-old ApoE^−/−^ mice, normoinsulinemic BATIRKO mice under high-fat diet and 52-week-old BATIRKO mice with lower UCP-2 levels had higher iNOS levels and higher vascular damage. In addition, anti-TNF-α antibody pre-treatment reduced iNOS expression, restoring UCP-2 levels, and improving vascular alterations from 52-week-old BATIRKO mice [24].

## Conclusions

In conclusion, our results suggest that insulin and TNF-α share an antagonistic effect on UCP-2 expression levels in vascular cells and also in the aorta in vivo. Thus, moderate hyperinsulinemia in response to insulin resistance or lowering of TNF-α levels within the aorta attenuates vascular damage, this protective effect being mediated by UCP-2 expression levels through iNOS.

## Additional files

Additional file 1: Figure S1. UCP-2 protein expression *in vivo*. UCP-2 protein levels were detected by Western blot and α-tubulin was used as loading control. UCP-2 protein levels in aorta artery from Control and ApoE^-/-^ mice at 24 weeks of age (A), Control, moderate hyperinsulinemic obese BATIRKO and normoinsulinemic obese BATIRKO under HFD (B), Control at 33 weeks of age, Control and BATIRKO and 52 weeks of age (C) and anti-TNF-α treated BATIRKO mice at 52 weeks of age (D). (A) Control at 24 weeks of age (C24; n=3); ApoE^-/-^ mice at 24 weeks of age (ApoE^-/-^24, n=3); *p<0.05 vs. C24. (B) Control under HFD (C; n=3); moderate hyperinsulinemic obese BATIRKO mice (BMH, n=3); normoinsulinemic obese BATIRKO mice (BN, n=3). †p<0.05 vs. BMH mice. (C and D) Control 33 weeks (C33, n=3); Control 52 weeks (C52, n=6); BATIRKO 52 weeks (B52w, n=5); BATIRKO 52 weeks + anti-TNF-α (B52+anti-TNF-α, n=3). †p<0.05 vs. C52; #p<0.05 vs. B52. Additional file 2: Figure S2. Characterization of vascular damage in BATIRKO mice under high-fat diet. (A) Vascular function was studied in aortic rings from control and BATIRKO mice under HFD. We evaluated endothelium-dependent relaxations to acetylcholine (Ach; 10^-5^ mol/L) and endothelium-independent relaxations induced by sodium nitroprusside (SNP; 10^-7^ mol/L) in phenylephrine (10^-6^ mol/L) precontracted rings. (B) Contractile responses to thromboxane A2 analogue (U46619; 10^-6^ mol/L) and Angiotensin II (Ang II, 10^-6^ mol/L) in aortic rings from controls and BATIRKO mice. (C) (Left) Representative photomicrographs of macrophages immunohistochemistry (F4/80) (first file of panels), of nitrotyrosine levels by immunohistochemistry (IHC) (second file of panels) and of superoxide anion levels in situ by hydroethidine method (third file of panels) in aortic roots from control and BATIRKO mice. (Right) Quantitative analysis of lesion area, macrophage infiltration, superoxide levels and of nitrotyrosine expression in aortic roots from control and BATIRKO mice. Results are expressed as mean ± SEM. Control (n=12), BATIRKO MH (n=6), BATIRKO N (n=10). *p<0.05 vs. Control; †p<0.05 vs. BATIRKO MH mice.

## Abbreviations

Ach: Acetylcholine
Ang II: Angiotensin II
Akt: Protein kinase B (Pkb)
ApoE^−/−^ mice: Apolipoprotein E knockout mice
BAT: Brown adipose tissue
BATIRKO: BAT-specific IR knockout mice
BATIRKO MH: Moderate hyperinsulinemic obese BATIRKO mice
BATIRKO N: Normoinsulinemic obese BATIRKO mice
ECs: Endothelial cell lines
L-NAME: Nitro-L-arginine methyl ester hydrochloride- NOS inhibitor
HFD: High-fat diet
IGF-1: Insulin-like growth factor-1
iNOS: Inducible nitric oxide synthase
IR: Insulin receptor
NF-κB: Nuclear factor kappa B
ROS: Reactive oxygen species
SNP: Sodium nitroprusside
STD: Standard diet
TNF-α: Tumor necrosis factor alpha
TXA2: Thromboxane A2
UCP-2: Uncoupling protein 2
U46619: TXA2 analogue
VSMCs: Vascular smooth muscle cells
WAT: White adipose tissue
wk: week
