# Supplementary figures and images for: Antagonistic effect of TNF-alpha and insulin on uncoupling protein 2 (UCP-2) expression and vascular damage
Source: Cardiovasc Diabetol. 2014 Jul 31;13:108. doi: 10.1186/s12933-014-0108-9 (PMC4149264; doi:10.1186/s12933-014-0108-9)

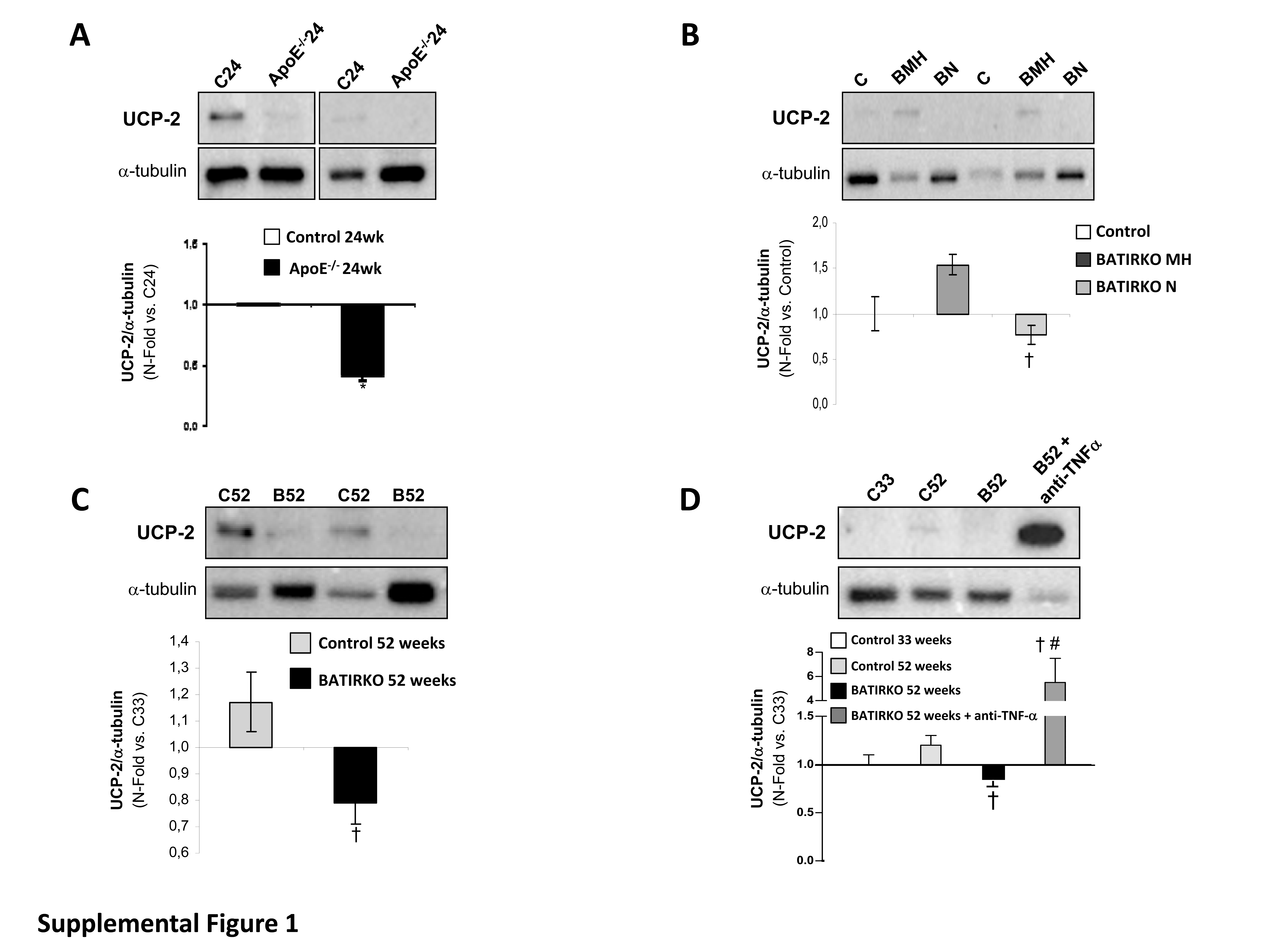

Supplement: Additional file 1: Figure S1. — UCP-2 protein expression in vivo. UCP-2 protein levels were detected by Western blot and α-tubulin was used as loading control. UCP-2 protein levels in aorta artery from Control and ApoE-/- mice at 24 weeks of age (A), Control, moderate hyperinsulinemic obese BATIRKO and normoinsulinemic obese BATIRKO under HFD (B), Control at 33 weeks of age, Control and BATIRKO and 52 weeks of age (C) and anti-TNF-α treated BATIRKO mice at 52 weeks of age (D). (A) Control at 24 weeks of age (C24; n=3); ApoE-/- mice at 24 weeks of age (ApoE-/-24, n=3); *p<0.05 vs. C24. (B) Control under HFD (C; n=3); moderate hyperinsulinemic obese BATIRKO mice (BMH, n=3); normoinsulinemic obese BATIRKO mice (BN, n=3). †p<0.05 vs. BMH mice. (C and D) Control 33 weeks (C33, n=3); Control 52 weeks (C52, n=6); BATIRKO 52 weeks (B52w, n=5); BATIRKO 52 weeks + anti-TNF-α (B52+anti-TNF-α, n=3). †p<0.05 vs. C52; #p<0.05 vs. B52. [file 12933_2014_108_MOESM1_ESM.tiff]

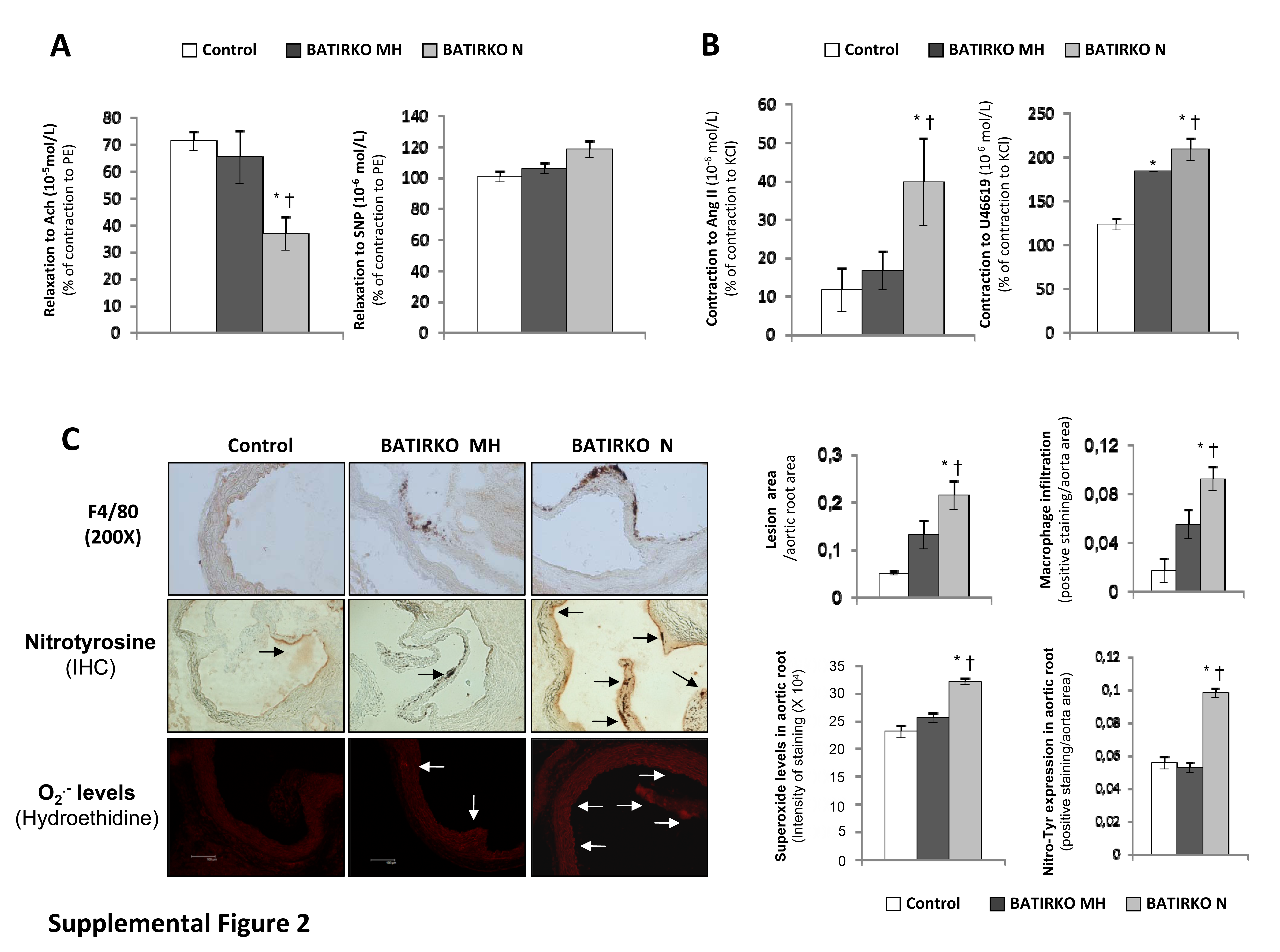

Supplement: Additional file 2: Figure S2. — Characterization of vascular damage in BATIRKO mice under high-fat diet. (A) Vascular function was studied in aortic rings from control and BATIRKO mice under HFD. We evaluated endothelium-dependent relaxations to acetylcholine (Ach; 10-5 mol/L) and endothelium-independent relaxations induced by sodium nitroprusside (SNP; 10-7 mol/L) in phenylephrine (10-6 mol/L) precontracted rings. (B) Contractile responses to thromboxane A2 analogue (U46619; 10-6 mol/L) and Angiotensin II (Ang II, 10-6 mol/L) in aortic rings from controls and BATIRKO mice. (C) (Left) Representative photomicrographs of macrophages immunohistochemistry (F4/80) (first file of panels), of nitrotyrosine levels by immunohistochemistry (IHC) (second file of panels) and of superoxide anion levels in situ by hydroethidine method (third file of panels) in aortic roots from control and BATIRKO mice. (Right) Quantitative analysis of lesion area, macrophage infiltration, superoxide levels and of nitrotyrosine expression in aortic roots from control and BATIRKO mice. Results are expressed as mean ± SEM. Control (n=12), BATIRKO MH (n=6), BATIRKO N (n=10). *p<0.05 vs. Control; †p<0.05 vs. BATIRKO MH mice. [file 12933_2014_108_MOESM2_ESM.tiff]
